# Supplementary material for: Event detection in football: Improving the reliability of match analysis
Source: PLoS One. 2024 Apr 18;19(4):e0298107. doi: 10.1371/journal.pone.0298107 (PMC11025972; doi:10.1371/journal.pone.0298107)
Supplement: Attachment — Submitted filename: rebuttal_letter.pdf [file pone.0298107.s001.pdf]

Dear editors, dear reviewers,

I wish to express my sincere thanks to you for taking the time and consideration to review our work. The review process has raised valid issues which we tried to address and improve our manuscript accordingly. Below is our response to each of the points that have been raised.

Include the problem statement, objective, motivation, and paper organization in the introduction section.

- Extended the introduction accordingly.

List out the strengths, limitations, and techniques of recent related existing methods in the related work section.

- Added detail about the methods employed in previous studies.

Provide a detailed analysis of the datasets with their data details, feature details, and volume of datasets.

- Added details about the data sets, in particular details about relevant features and the number of relevant events in the training and test sets.

This work is not compared with any existing methods. The authors should provide any statistical, mathematical, or comparative proof to claim that this work is state-of-the-art.

- Added a discussion of time and space complexity, and a more explicit comparison of detection performance with previous results.

Include findings, strengths, limitations, application area, and future work of the proposed method in the conclusion section.

- Added details to the conclusion section.

1. Authors must show explain the novel contribution of the work with proper justification of the outcomes. What novelty is established in this work compared to existing works?

- Added some clarity concerning our contributions throughout the paper. The novel contributions have been listed at the end of the introduction.

2. The computational complexity in terms of time and space must be discussed. Also, compare the proposed method in terms of computational complexity?

- Added time and space complexity. All rule-based event detection algorithms should trivially be of linear complexity as events are tightly localized within in the positional data.

3. Literature survey need to be updated based on current state of art methods. Some more paper based on Event detection in football:.

- We decided to limit ourselves to studies that operate upon positional data and believe our selection of such studies should be complete. In the case that we missed scientific work on that specific topic, we would be happy to learn about it and add it to the paper! We did not explicitly include papers where events are detected directly from video due to the large

categorical differences between those two approaches, especially regarding their main goals and areas of application (comprehensive industry-scale automation vs. research purposes and internal model building in clubs/federations), as touched upon in the introduction section. A paragraph about the practical role of the proposed algorithm has been added in the conclusion.

4. The abstract need to be improved and the outcome of the work in terms of achieved various other performance calculations must be included in the abstract.

- Added more performance calculations in the abstract, mentioning the outcomes more explicitly.

5. Explaining the problem and the gaps in existing literature in a concise but self-contained way (although readers might wish to consult references, they should not be forced to do so)

- Added more details to the literature background and pointed out the research gap more clearly.

6. Organization of the paper can be added at the end of introductions.

- Added a corresponding paragraph.

7. Comparative analysis of various performance parameters with respect to sate of art methods must be discussed. More recent state-of-the-art approaches should be compared; the experiments should use more sizable real-world data sets from public repositories (if any);

- Added a more explicit comparison of performance parameters with the state of the art and mentioned the size of the data sets as a limitation. While larger data sets would certainly be beneficial, those are not available to us due to the proprietary nature of most football match data. I also added a table with an overview of the number of reference events for each of the four data sets to provide a more explicit impression of our sample sizes.

8. Add industrial significance of the proposed approach.

- Added a paragraph in the conclusion section.

9. Results must be verified some more other data sets also. Describing in detail the data set used and what are the expected outcomes- widening the experimental comparison including other data and methods.

- Added detail the data used. More data is not available due to the proprietary nature of football match data.

10. Comparative analysis of various performance parameters with respect to various data sets must be discussed. The comparison can be a bit unfair if different data is not used for comparative analysis.

- Completed the comparison such that each comment in the results section includes all the analysed data sets without leaving out any single one.

11. Limitations of the proposed work must be included.

- Added a paragraph in the conclusion discussing limitations.

12. Precision vs. recall curves of the proposed algorithms with respect to different data sets must be included.

- Since not all classifiers are probabilistic, we decided to use F1-scores to allow for a comparison between classifiers.

13. Implementation platforms with complete specifications of the system must be included.

- Added information about the software framework used for implementation. Hardware specifics would not be informative since the code is executable on any regular computer with a compatible Python environment and sufficient RAM.

14. How much data should be considered for training and testing for model implementation? Details of training and testing data sets must be tabulated.

- Added more information about the training and test sets in tabular form.

14. To make the proposed algorithm of this article more readable use pseudo-code.

- Added pseudo-code.

15. In all results tables'/figures utilized datasets like in table 2, 3 and 4 etc. must be cited with proper and specific citations.

- Added the suggested citations in all tables.

16. Various visualized results based on proposed work must be added and also compared the results with existing work.

- Added a more explicit performance comparison in the discussion section.

17. Comparative analysis with respect to real-time time analysis is missing?

- Added a corresponding part in the discussion section.

If there remain any issues with our revised manuscript, we would be happy to address those!

Best regards

Jonas Bischofberger, corresponding author
